# Good Arts, Good Mental Health^®^: the effectiveness of an Australian health promotion media campaign in promoting community mental wellbeing via the arts

**DOI:** 10.3389/fpubh.2025.1594846

**Published:** 2025-06-26

**Authors:** Christina R. Davies, Melanie T. Pescud, Rhonda Clifford, Richard McGrath, Annie Thomson, Mia Jeffrey, Melissa Stoneham, Terri Pikora, Peter Wright, Sonya Girdler, Loretta Baldassar, Stephen Clift

**Affiliations:** ^1^Centre for Arts, Mental Health and Wellbeing, School of Allied Health & School of Humanities, The University of Western Australia, Crawley, WA, Australia; ^2^School of Allied Health, University of Western Australia, Perth, WA, Australia; ^3^Allied Health and Human Performance, University of South Australia, Adelaide, SA, Australia; ^4^Public Health Advocacy Institute, Curtin University, Bentley, WA, Australia; ^5^School of Education, College of Health and Education, Murdoch University, Murdoch, WA, Australia; ^6^Curtin Autism Research Group, School of Allied Health, Curtin University, Bentley, WA, Australia; ^7^School of Arts and Humanities, Edith Cowan University, Joondalup, WA, Australia; ^8^Sidney De Haan Research Centre for Arts and Health, Canterbury Christ Church University, Canterbury, United Kingdom

**Keywords:** health promotion, mental health, arts, campaign, Good Arts Good Mental Health, dose

## Abstract

**Introduction:**

This study describes and evaluates *Good Arts, Good Mental Health*^®^ (GAGMH), a groundbreaking, population-level, arts-mental health promotion media campaign. The objectives of the campaign (Wave 1) were to increase brand awareness, comprehension, and agreement with the tagline *Good Arts, Good Mental Health*^®^ and empower the general population to form an intention to engage in the Arts for their mental wellbeing.

**Methods:**

The campaign ran from August to September 2024 (4 weeks), cost AUD$198,965 (23% creative and 77% media/advertising distribution), and targeted the Western Australian (WA) general population aged 18–65 years, all genders, in both metropolitan and regional areas. The campaign was distributed through a variety of platforms, channels, visual, audio, and static assets. To gauge the success of the campaign, a process evaluation and (short-term) outcome evaluation were conducted by sourcing online analytics and conducting an online survey of the campaign target group (*n* = 661).

**Results:**

Overall, campaign reach and frequency were optimal and met set targets. Campaign website engagement substantially increased from baseline (7,505 to 53,810 events 1 month after the campaign). Advertising cost-per-reach was effective and ranged from $0 for free/organic media to $0.10 for radio. For paid media channels, the highest reach (948,106 people) and best cost-per-reach ($0.02) were delivered by Meta (Facebook and Instagram).

Measured as both (1) a proportion of total respondents and (2) a sub-set analysis of each preceding level in the cognitive impact hierarchy, post-campaign, one-in-four respondents were aware of *Good Arts, Good Mental Health*^®^. This is comparatively high for a new tagline and health campaign without TV advertising. Overall, comprehension was satisfactory. Agreement and intention to act on the message were high and double that of comparison Western Australian health promotion campaigns.

**Discussion:**

Study findings indicate the GAGMH campaign was successful. If funded, future waves of the campaign could build on Wave 1 to reinforce message awareness, increase understanding of GAGMH concepts, focus more on the *arts-mental health dose*, and extend the outcome evaluation by measuring behavioral action. The information contained in this study is useful to Public Health, Mental Health, and Arts-Health professionals in the planning, implementation, and evaluation of future arts-mental health promotion strategies and campaigns.

## Introduction

Do you take part in recreational arts or sport for better mental health? How do you “know” sport is good for your mental health, and why do you (try to) exercise for 30 minutes (1) per day? Part of the reason is because the evidence and benefits of sport and physical activity are widely promoted by government and health organizations. Underpinned by both social marketing (2) and communication-behavior change models, (3) there are many examples of population-level, health promotion media campaigns that encourage engagement in recreational sport and active living including “Be Positive, Be Connected, Be Active,” (4) “Find 30,” (1, 5) “Make Healthy Normal,” (6) and “Life. Be in it,” (7) In comparison to sport, however, recreational arts are under-promoted by government and health organizations, especially in terms of health promotion media campaigns, sponsorships, resources, learning, and engagement opportunities (8–12).

Globally, mental health issues are increasing for reasons including the growing climate crisis, disease outbreaks, health, social, and economic inequities (13, 14). Similar to engagement in recreational sport, there is strong evidence that recreational arts engagement enhances mental wellbeing (15–20) and should be utilized as a mental health prevention and early intervention strategy (15). An *arts-mental health dose* (i.e., how much arts engagement is needed for good mental health) has also been calculated, with two or more hours of recreational arts engagement per week linked to better mental wellbeing than lower levels of engagement for adults in the general population (10). The purpose, therefore, of this paper was to extend the field by describing and evaluating a world first, trademarked, arts-mental health promotion media campaign *Good Arts, Good Mental Health*^®^ (GAGMH) that aimed to address this lack of arts-mental health promotion at a population level. Good mental health enables individuals to contribute to their community, realize their potential, work productively, and cope with the stresses of everyday life (21). Good mental health is essential for individual and community wellbeing (22). In Australia, poor mental health is a leading cause of disease burden, injury, and disability, with one in five adults experiencing mental illness each year (22, 23). As a result, the Australian health system is under increasing pressure to deliver mental health services which in 2021–2022 was estimated to cost AUD$12 billion (24, 25). Evidence-based, population-level innovation, such as *Good Arts, Good Mental Health*^®^ is needed to address the mental health crisis Australia and the world are facing.

### Recreational arts engagement

**Figure 1:**
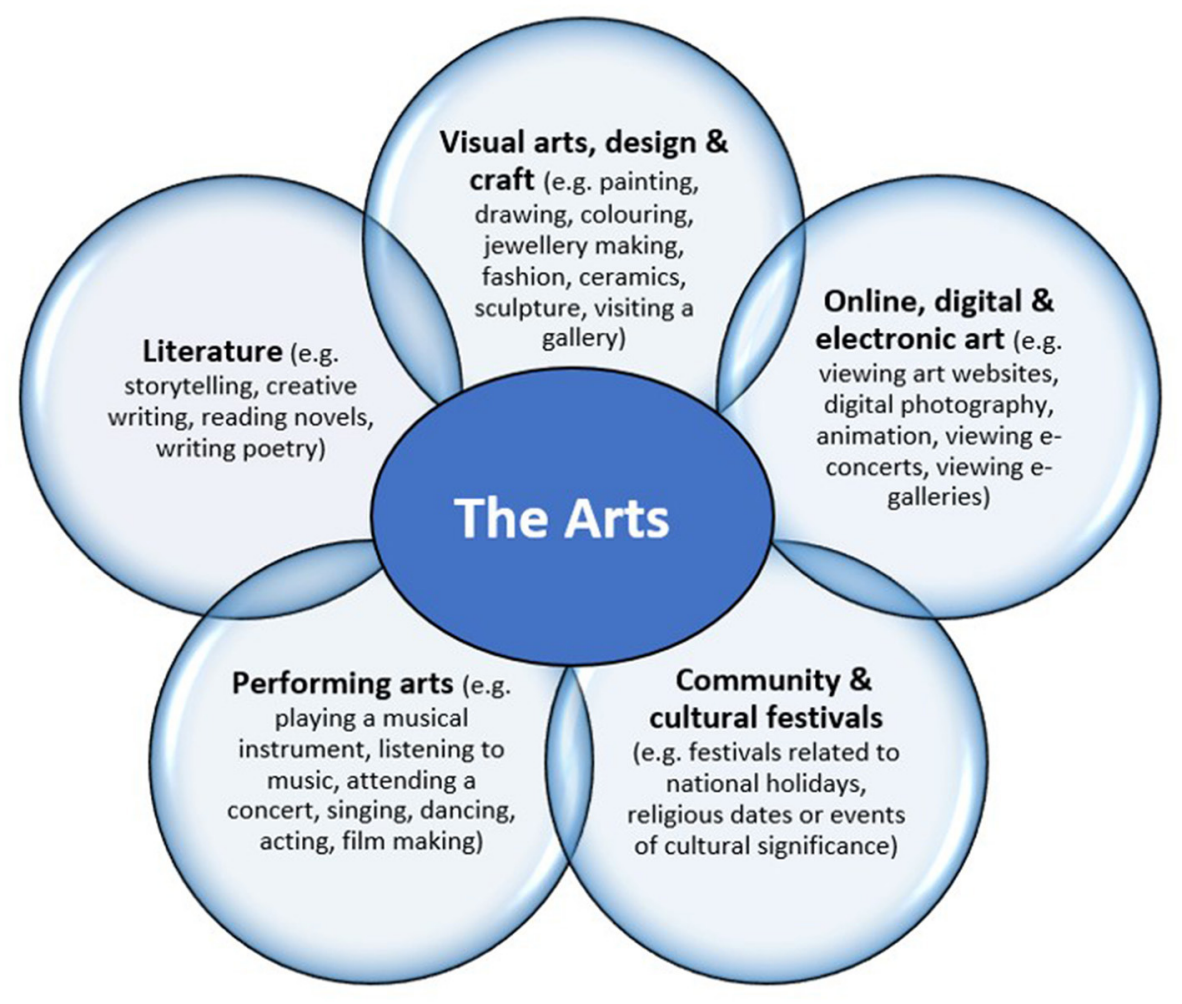
Recreational arts—art forms and activities (re-printed with author permission) (26, 27).

*Recreational arts engagement* is an “umbrella term” encompassing the various ways in which people engage in the arts in their everyday life (e.g., listening to music, reading books, coloring, photography, sewing, going to concerts, festivals, art class, and so much more) (26). Within this definition, *the Arts* has been defined by Davies et al. (26, 27) through five art forms and a variety of activities and events, i.e., visual arts, design and craft; community and cultural festivals; online, digital and electronic arts; performing arts, and literature (Figure 1). For clarity, it should be noted that recreational arts engagement (the focus of this study) and art therapy/therapies are not the same thing (26). Art therapy/therapies involve a therapeutic relationship between a qualified therapist and individual who undertake creative activities for diagnostic or treatment purposes (26, 28). Recreational arts engagement, however, is something anyone can do, regardless of health status, as part of their everyday life, for enjoyment, entertainment, socially, or as a hobby (26). Recreational arts engagement occurs on a continuum from active involvement (making) to receptive involvement (attending, listening, and viewing) and occurs within a variety of settings including the home, work, schools, parks, community centers, cultural centers, etc (15, 26, 29). Recreational arts can be undertaken alone (as an individual) or with others (e.g., friends, family, artists, musicians, audience members, and art class participants) (26). In Australia, the most popular modes of recreational arts engagement are listening to music (97%), reading books (79%), and attending arts events (72%) (30). Even though yearly engagement in the arts is high (31), most adults engage (dose-response) at levels insufficient to achieve mental wellbeing benefits (15), that is, less than the estimated *arts-mental health dose* of two or more hours per week (10).

### Public health media campaigns

*Population-level health communication* refers to the various means by which public health information reaches a large number of people (32). Media campaigns are a form of paid advertising where a complex message is communicated in terms of information and imagery (33, 34). Public health media campaigns play a pivotal role in population-level health communication to promote, maintain, and improve population health (35) and are used/have been used to influence community beliefs and behavior around a number of health issues including immunization, seat-belt use, alcohol consumption, sun safety, physical activity, healthy eating, and tobacco use (1, 6, 34, 36–39). Public health media campaigns involve a variety of channels including television, radio, cinema, print media (newspapers, magazines, etc.), websites, signage (posters, billboards, flags, etc.), social media (Instagram, Facebook, Twitter/X, LinkedIn, TikTok, etc.), online advertising (YouTube, Google, etc.), educational resources (brochures, flyers, and newsletters), mobile phone technology (SMS messaging, campaign Apps, advertising within gaming/mobile apps), campaign merchandise (bags, stickers, pens, stress-balls, hats, t-shirts, etc.), engagement opportunities (workshops, courses, e-learning, programs, etc.), and endorsements (by celebrities, community leaders, politicians, influencers, etc.) (32, 34, 37, 39, 40). In general, public health media campaigns often use messages/taglines as a cognitive strategy to influence thinking and the adoption of health-enhancing behaviors (34, 41). When delivering a campaign, message/tagline effectiveness is often assessed by measuring “*reach*” (number of people who saw the content), “*frequency*” (number of times a person was exposed to the content), cost, and cognitive impact of the message (i.e., awareness, comprehension, agreement, and intention) (3, 6, 37, 42–47). With regard to cognitive impact, underpinning the approach and evaluation of many Western Australian health promotion campaigns (5, 43, 44, 48–50), including GAGMH, is McGuire's communication-behavior change model or “communication-persuasion model” (3). McGuires model has been used extensively in mass-media campaigns and emphasizes a cognitive communication sequence which starts at message awareness and is followed by comprehension, agreement, and intention to act on the message (3). In Western Australian, physical activity (e.g., Find 30), anti-smoking (e.g., Quit), sun protection (e.g., Sun Smart), nutrition (e.g., Go for 2 and 5), and mental health (e.g., Act, Belong Commit) campaigns have been evaluated by calculating this communication sequence as a proportion of total respondents and also as a subset for each level within the cognitive impact hierarchy (5, 43, 44, 48–50).

### The Good Arts, Good Mental Health^®^ (GAGMH) initiative

Given the strong evidence that recreational arts engagement enhances mental wellbeing (15), it is time to more effectively utilize and promote this low cost/no cost, non-pharmacological method through a population-level, mental health promotion media campaign. The GAGMH initiative started in January 2022 and is led by arts-health research academics based at the University of Western Australia. GAGMH has been developed in partnership with the community, six universities, and 31 government, industry, and philanthropic partners. GAGMH is evidence-based, guided by the literature (3, 10, 15, 26, 27, 29, 37, 46, 51–61), multi-award winning, and aims to improve community mental wellbeing by communicating the value of recreational arts as a mental health promotion strategy. GAGMH is inclusive of all art forms and arts activities (26) and respectful of individual differences, individual preferences, budgets, lifestyle, and life-stage factors. Where possible, the GAGMH campaign website (www.goodartsgoodmentalhealth.com.au) and social media accounts (Instagram and Facebook handle = @goodartsgoodmentalhealth) offer low-cost and no-cost options for engagement (e.g., open-access to publications, free learning videos, downloadable resources, coloring sheets, a Spotify playlist, etc). GAGMH also promotes the arts-mental wellbeing relationship to the general population via a range of learning (e.g., face-to-face workshops, professional development, and e-learning) and demonstration programs (large, medium, and small events). Most recently, GAGMH launched a world first, population-level, trademarked, arts-mental health promotion media campaign, *Good Arts, Good Mental Health*^®^ which is the focus of this paper.

#### The GAGMH media campaign^®^ (Wave1)

The GAGMH campaign (Wave 1) was promoted across the whole of the Australian state of Western Australia (WA) from the 17 August to the 14 September, 2024. WA is Australia's largest state with a land area of 2.6 million square kilometers (i.e., WA is larger than Western Europe, 10 times the size of the UK, and 4 times the size of Texas) (62) and has a population of ~2.9 million people (63). The GAGMH campaign target group was adults, 18–65 years, all genders, in both metropolitan and regional areas of WA. Guided by theories of positive psychology, social psychology (e.g., Theory of Planned Behavior), social epidemiology (psychosocial and eco-social), social marketing, and communication-behavior change (2, 3, 64–66), the primary focus of the GAGMH campaign was to (Objective 1) increase brand recognition/awareness, comprehension, and agreement with the tagline “*Good Arts, Good Mental Health”* and (Objective 2) empower community members to form an intention to proactively and regularly engage in the arts activities that make them feel good, therefore enhancing their mental wellbeing.

The creative components of the campaign (e.g., wording, images, and language) were co-designed via a rigorous formative research process including surveys, focus groups, and interviews with project partners, i.e., community members *n* = 3,560, industry *n* = 29, a state government reference group (from arts, mental health, and health departments) who met with GAGMH on a quarterly basis *n* = 7, philanthropy who met with GAGMH on a quarterly basis *n* = 2, and a community/consumer reference group with backgrounds in the arts, public health, and/or lived experience of mental health challenges/recovery, *n* = 6. Project partners, especially community members, emphasized the importance of using positive and inclusive images (all genders, ages, abilities, ethnicities, etc.), positive words (e.g., good mental health, mental wellbeing, happy, strong, relaxed, connected vs. stressed, anxiety, depressed, and lonely), and invitational words (e.g., try vs. start, as the general population did not wish to be told what to do) and preferred the word “arts” to “creative” or “creativity,” as to them, creativity implied making and some people only wanted to engage receptively in the arts (e.g., attend, view, listen, and read). Project partners also recommended promoting a range of arts activities, low-cost and no-cost options, easily actionable tips, and suggestions in non-academic, everyday language. As above, the partner-endorsed campaign *tagline* was *Good Arts, Good Mental Health*^®^. The *manifesto* was as follows:

*You don't have to be good at art for the arts to be good for you. Do the art that makes YOU feel GOOD. TRY for two hours per week. It could make a difference to your mental health. Good Arts, Good Mental Health”.*

The tagline and manifesto were positive and solution-focused and encouraged people to think about and then take part in the arts activities that made them feel good (vs. sad, embarrassed, stressed, not interested in). It acknowledged that people enjoy different arts activities (e.g., one person may like singing, another may prefer attending concerts and knitting, while another may prefer reading, dancing, and painting). The manifesto also aimed to empower people with the knowledge that the arts could positively impact their mental health and that they could take part, no matter their skill level. While not the primary focus of Wave 1 of the campaign, the *arts-mental health dose* of two or more hours per week was also introduced to the community.

#### Campaign assets and delivery

Utilizing the creative ideas developed with our project partners, a professional communication agency was employed to advise and support the campaign (i.e., asset creation, media deployment and delivery, and collection of process evaluation data). The campaign budget was AUD$198,965, of which 23% was used to create the website, visual, audio, and static assets and 77% for media distribution. As described below, a variety of campaign assets were created and delivered; however, paid television advertising was not possible due to budget constraints.

**Figure 2:**
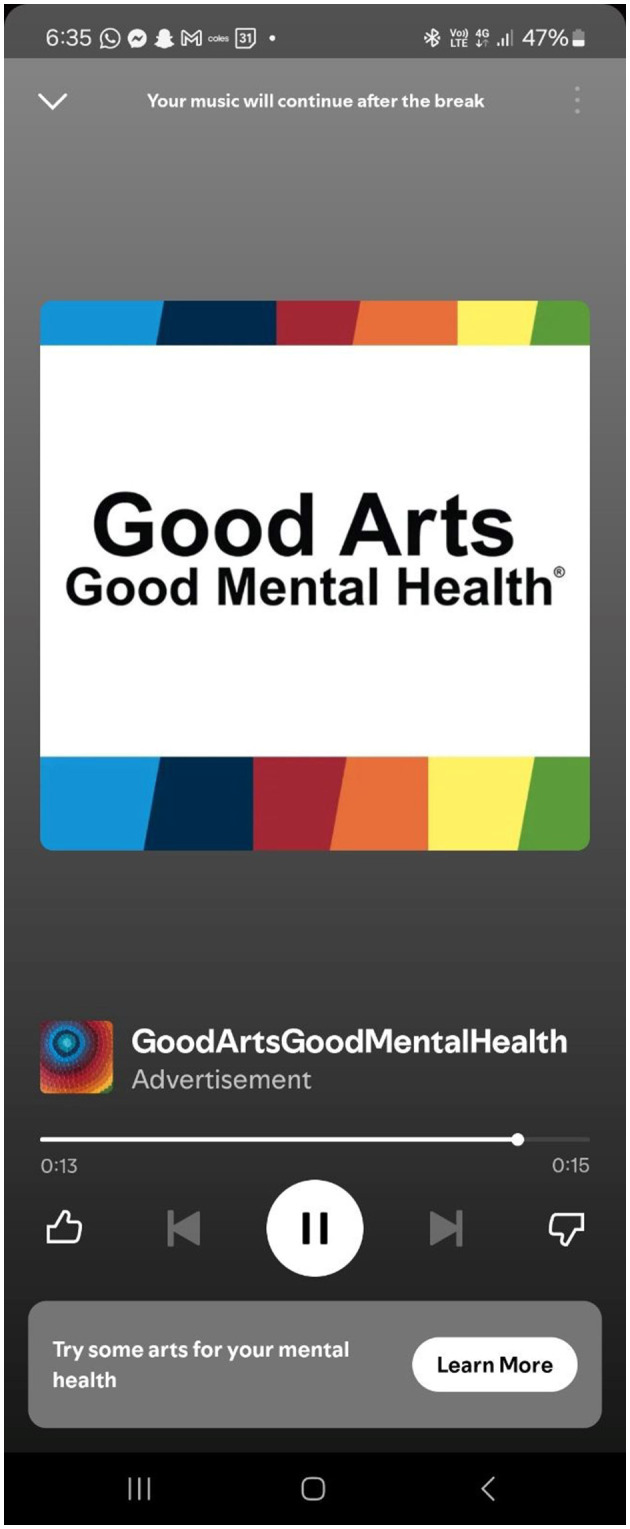
Spotify advert.

**Figure 3:**
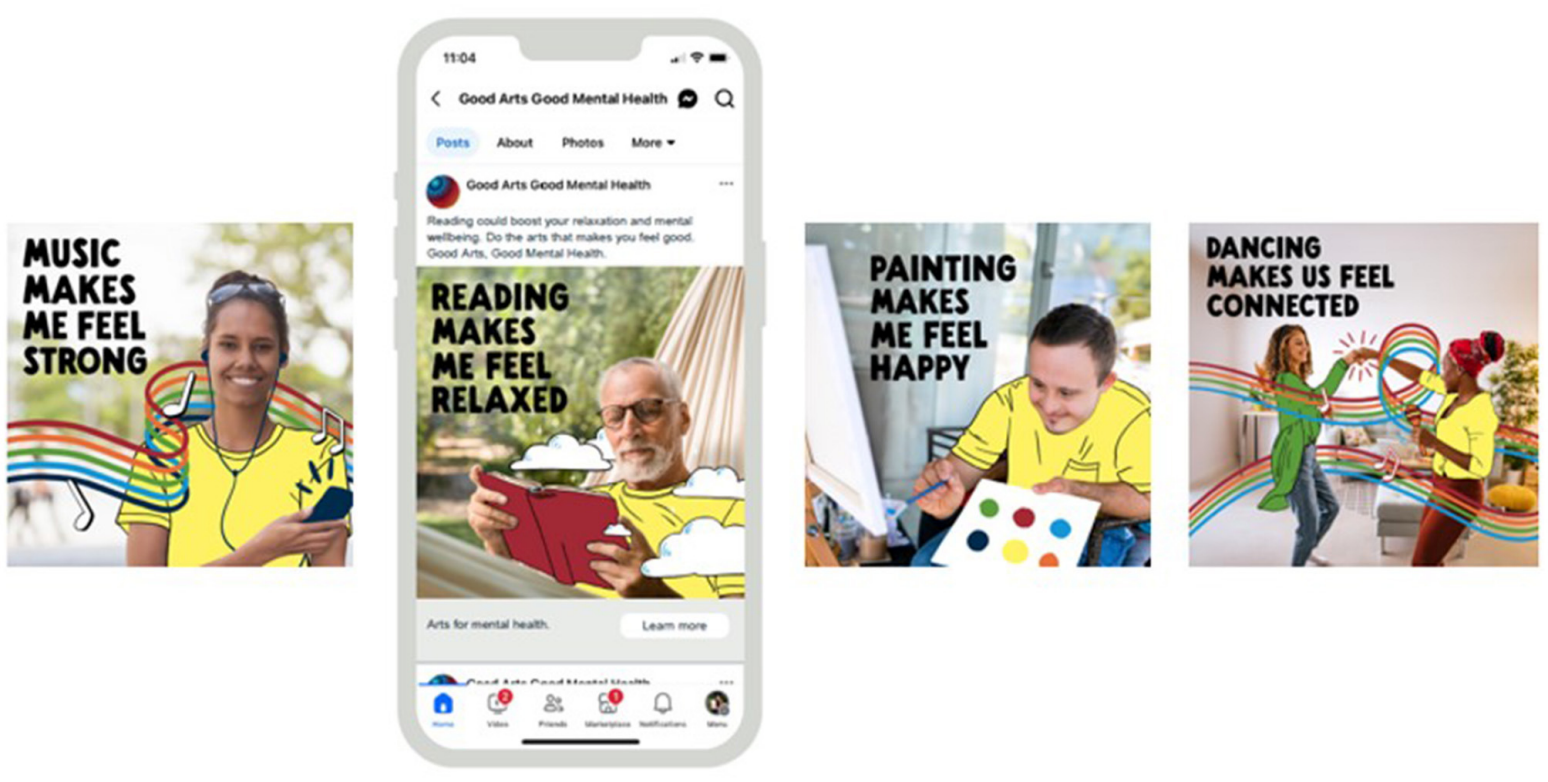
GAGMH Meta tiles.

**Figure 4:**
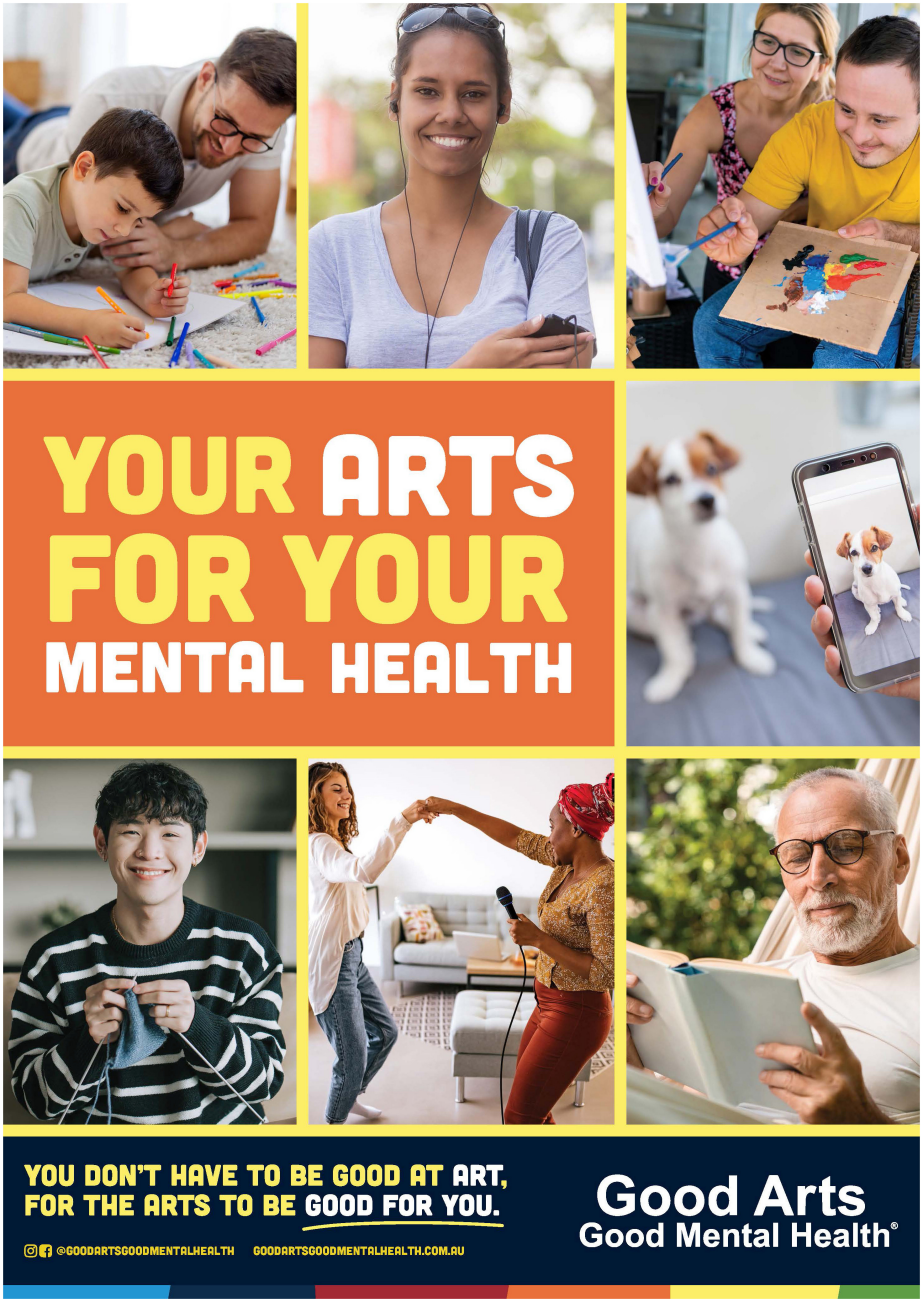
GAGMH poster.

1. **Radio advertising:** A 15 second advertisement (advert) based on the manifesto was developed for radio. The script was: “*Music, reading, painting, dance - whatever art makes you feel good. You don't have to be good at art, for the arts to be good for you. Try for two hours per week*. *It could make a difference to your mental health. Good Arts, Good Mental Health*.” Due to their popularity and high prevalence of engagement by the community (30, 31, 67), music, reading, painting, and dance were said at the start of the advert to increase the possibility of immediate advert relevance to listeners. The script was spoken in a female voice (early 30s, friendly, happy, kind). The background music was relaxed and “easy-listening.” The radio advert was played by two metropolitan and 13 regional radio stations via broadcast and digital audio. A total of 3,463 advertising spots over 4 weeks were delivered (1,909 paid and 1,554 pro bono). Due to a partnership between one of the radio stations and a popular chain of supermarkets, the GAGMH advert was also played in 100 supermarkets across WA (30 spots per week for 4 weeks). The GAGMH radio adverts were complemented by co-branded radio station adverts, show/talent integration (live reads), competitions, prizes, GAGMH merchandise giveaways (e.g., GAGMH eco-bags, pens, coloring packs, etc), and social media posts/stories about the arts-mental health relationship from the radio station and station talent.
2. **Spotify advertising:** The same 15 second advert developed for radio was played on *Spotify Free*. The logo and banner were designed in the GAGMH font, brand colors and contained the campaign tagline. The advert also contained a tagline extension “*Try some arts for your mental health*” and a “*learn more*” button that jumped listeners to the campaign website (Figure 2). Spotify Ad Manager was used to distribute the paid GAGMH advert to the campaign target group.
3. **Meta advertising (Facebook and Instagram):** Similar to the radio and Spotify adverts, the focus for the paid Meta advertising was also music, reading, painting, and dance (Figure 3). For each arts activity, the following was developed: (i) one square and one long format illustrated tile, (ii) wording within the post (e.g., for the music tile the wording was “*Music could help you relax and lift your mood. Do the arts that make you feel good. Good Arts, Good Mental Health*”), (iii) an arts-mental health image headline (e.g., for dancing, the headline was “*Dancing makes us feel connected*”), and (iv) a post headline “*Arts for mental health*” and “*learn more*” button that jumped viewers to the campaign website. Meta Ad Manager was used to promote the paid posts to the target group as a single image and/or carousel of images.
4. **Editorial:** A popular local news/media service was paid to write an article about GAGMH. The company promoted their article through its e-newsletter which was delivered to 95,583 Western Australian subscribers. The news service was also paid to create a short GAGMH social media video/reel which they shared through their Instagram, Facebook, LinkedIn, and TikTok accounts.
5. **Campaign website:** A web consultant was paid to design and build the campaign website www.goodartsgoodmentalhealth.com.au. The website contained a home/landing page (welcome video, sub-hub intro, social media intro, and partner/advisor/mentor acknowledgment) and five sub-hubs titled *Learning* (learning videos, quiz), *Research* (information about the GAGMH team, open-access to papers, reports, publications), *Campaign* (free access to a 5-day arts challenge, poster, Spotify playlist, links, and downloadable resources), *News* (videos and articles), and *Youth/Kids* (free activities, coloring sheets, links, and downloadable resources). The website also linked to the GAGMH social media accounts and gave viewers the option to sign-up to the GAGMH newsletter. The website was launched on 25 July 2024 to ensure that any website problems or errors were rectified before the campaign launch.
6. **Signage:** A “static” poster in the GAGMH font and brand colors that highlighted the popular arts activities of music, reading, painting, and dance but also coloring, photography, and knitting was created (Figure 4). The poster included the campaign tagline, manifesto suggestion “*You don't have to be good at art, for the arts to be good for you*” and a tagline extension “*Your arts for your mental health*” to increase viewer comprehension and understanding of the message/images. The poster was printed in color on 150GSM gloss paper (*n* = 750 in A4, A3). The poster was distributed electronically or in hard copy to project partners, metropolitan and regional arts organizations, and local governments so that they could be placed in high visibility, community relevant areas. In the metropolitan area, the poster was also displayed in 300 cafes, community venues, and notice boards by a signage distribution company.
7. **Organic media:** In addition to the “paid assets” described above, the campaign was also amplified by organic or free media. This included a media release which resulted in 12 newspaper/magazine/e-articles. The first author was also invited to conduct a national mid-day TV interview with one of Australia's leading TV stations and 14 radio interviews. In addition, the campaign was promoted in three GAGMH e-newsletters to 544 subscribers (with newsletter sharing requested), in eight project partner/community newsletters, 73 Instagram posts, 53 Facebook posts, 26 Linked-In posts, 31 Twitter posts, and eight YouTube videos (organic media reach during the campaign is unknown).

As stated above, the purpose of this paper is to describe the *Good Arts, Good Mental Health*^®^ campaign (see above). The remainder of this paper will evaluate the reach, frequency, cost, and cognitive impact (awareness, comprehension, agreement, and intention) of the GAGMH campaign.

## Method

Permission to conduct this initiative and evaluation was granted by the University of Western Australia Human Research Ethics Committee (2022/ET000140).

### Process evaluation

A process evaluation was conducted to determine the success of the campaign implementation. Prior to the campaign starting (25 July−16 August 2024), during the campaign (17 August−14 September), and 1 month after the campaign (14 October), users and event information were collected via Google Analytics for the campaign website (e.g., page views and downloads). From 17 August to 14 September 2024 (during the campaign), reach and frequency analytics were sourced about campaign channels/platforms from Spotify Ad Manager, Meta Ad Manager, Campaign Manager 360, service providers, and Google Analytics. According to the literature, it can be estimated that for a spend of ~$100,000, the level of advertising intensity needed to drive optimal population-level brand awareness occurs at a reach of between 36% and 46% and frequency of two to five exposures. (68–72) Given this, our GAGMH goal was to achieve a reach of 36%−46% and frequency between two and five exposures. “Cost-per-reach” (i.e., $cost divided by the number of individuals who saw the content) was also calculated for each campaign channel/platform as a measure of effectiveness. A “good” cost-per-reach is suggested to be between $0.20 and $1.00 (73).

### Outcome evaluation (short-term)

To determine whether the campaign increased *awareness, comprehension*, and *agreement* with the tagline *Good Arts, Good Mental Health*^®^, and encouraged community members to form an *intention* to engage in the arts for their mental wellbeing, a (short term) outcome evaluation was conducted. An independent market research company was contracted to collect the evaluation data. From 14th to 23rd September 2024 (after the campaign), an online survey was conducted with members of the campaign target group who were randomly selected from the market research companies panel of 40,000 Western Australians. For precision purposes, a minimum sample size of 385 surveys was needed to achieve 80% power at the 5% level of significance. The evaluation survey was based on previously established surveys (50, 74) and asked three demographic (i.e., gender, age group, and location) and five cognitive impact questions (two awareness and one comprehension, agreement, and intention question).

To measure awareness, each respondent was asked whether they “*recalled seeing or hearing any health messages related to taking part in the arts for good mental health*” (unprompted awareness), followed by if they had “*seen or heard the message Good Arts, Good Mental Health*^®^” (prompted awareness). Respondents were then asked, “*What do you think the Good Arts, Good Mental Health*^®^ *message means?*” (comprehension). As this was the first time the media campaign had been run, all respondents were then informed, “*Good Arts, Good Mental Health*^®^ *aims to encourage people to do arts activities that make them feel good for better mental wellbeing. This includes reading books, listening to music, playing a musical instrument, singing, dancing, going to a concert, woodwork, craft, sewing, pottery, painting, creative writing, coloring, and so much more*.” Respondents were then asked, “*Do you agree with the Good Arts, Good Mental Health*^®^ *message*?” (acceptance/agreement) and “*Does knowing about the Good Arts, Good Mental Health*^®^ *message, make you think about doing something related to the message?*” (intention). As a comparative benchmark, Australian campaigns have been found to achieve awareness levels between 4% and 98%, with an average of 58% (1, 6, 38, 48, 75, 76). As is the case with the GAGMH tagline, new health taglines with little to no TV advertising have been found to achieve message awareness of between 4% and 30% (the GAGMH awareness goal) (76). As a proportion of all respondents surveyed and as a sub-set of the behavioral cognitive hierarchy, on average, established WA health promotion campaigns (that include TV advertising) have been found to achieve comprehension levels of 74% (43% of total respondents surveyed), agreement of 92% (40% of total respondents surveyed), and intention to act of 41% (16% of total respondents surveyed) (48).

### Analysis

The number, reach, and frequency of process evaluation analytics are reported as sourced from Spotify Ad Manager, Meta Ad Manager, Campaign Manager 360, service providers, and Google Analytics by the communication agency. The cognitive impact data were analyzed using IBM SPSS Statistic (Version 29). The analysis involved a descriptive investigation of the data followed by a chi-square analysis and pairwise comparisons using a Fisher's exact test to check for demographic differences. For reasons of transparency, respondent awareness, comprehension, agreement, and intention to act on the message were calculated as both a proportion of total respondents (*n* = 661) and a proportion of each preceding level in the cognitive impact hierarchy. The question about agreement asked for a yes/no response. The comprehension question was open-ended and coded as “correct” if respondents mentioned taking part in the arts activities that made them, or people in general feel good so as to promote, maintain, and/or improve their mental health/mental wellbeing. Respondents needed to mention both (1) the arts—in general or specific recreational arts activities, and (2) mental wellbeing outcomes, e.g., “*Doing art can be good for mental health,”* “*Do arts and crafts to be happy*,” and “*Try arts for good mental health*.” Comprehension was coded as incorrect if the respondents reply was non-specific, they were unsure or did not know, e.g., “*good for all*” and “*not sure*.” Comprehension was also coded as incorrect if respondents did not mention both arts engagement and mental wellbeing (e.g., “*Doing arts*”) or mentioned “therapy,” “art therapy,” or mental illness (e.g., “*Art provides therapy for mental health challenges”)*. The awareness and intention questions asked for a yes/no response and if “yes,” respondents were asked to clarify which message(s) they saw or heard (awareness) and what they intended to do (intention). In this way, awareness and intention responses could be checked. It should be noted that intention could include responses related to the respondent (e.g., “*Listening to music makes me happy so I could do that*”) and also encouraging others to engage in the arts to promote, maintain, and/or improve their mental wellbeing (e.g., family, co-workers, and friends). To increase reliability, all open-ended responses were reviewed and coded independently by the second author and a research assistant. To increase inter-rater reliability, before coding commenced, coders took part in a training exercise with the first author to ensure coding consistency. Any coding differences or disagreements were moderated and resolved by the first author via discussion with both the second author and research assistant.

## Results

### Process evaluation

Advertising reach and frequency varied by campaign channel (Table 1). Overall, the advertising reach was between 33% and 80% of the Western Australian (WA) population, (i.e., 33% = 948,106 Meta reach divided by the 2.9 million WA population which assumes other channels/platform reach was not mutually exclusive; 80% = 2,325,833 each platform reach taken as mutually exclusive divided by the 2.9 million WA population). On average, our advertising frequency was 7.3 across channels, which is high. The advertising cost-per-reach ranged from $0 (free/organic media) to $0.10 (radio). For paid media channels, the highest reach (948,106 people) and cost-per-reach ($0.02) were delivered by Meta (Facebook, Instagram).

Table 1Process evaluation results^*^: GAGMH (Wave 1) reach, frequency, impressions, cost, and cost-per-reach by advertising channel/platform; Website users, events, and cost.

**Table:** 

**Advertising channel/platform**	**Reach (estimated number of people who actually saw the content)**	**Frequency (estimated number of times a person was exposed to the content)**	**Impressions (exposure—number of times the channel has displayed the content)**	**Media/Advertising distribution cost ($AUD)**	**Cost per reach**
Radio	873,815	14.6	11,242,000	$18,595 Regional $70,000 Metro	0.10
Spotify	218,957	7.8	1,700,400	$12,530	0.06
Meta	948,106	5.9	5,593,200	$18,330	0.02
Editorial	284,955	1.2	341,946	$14,906	0.05
Signage	–	–	–	$562	–
Organic media	–	–	–	$0 (no cost)	0.00
Agency fees, licenses	NA	NA	NA	$17,574	NA

**Table:** 

**Website**	**4 weeks prior to the campaign**	**During the campaign (17 August-14 September)**	**1 month after the campaign**	**Website cost ($AUD)**
Users	614	6,722	7,940	$29,870
Total events	7,505	35,879	53,810	

* WA population = 2.9 million people (63), campaign target group = WA adults 18–65 years, all genders, metropolitan, and regional areas; total cost = $198,965; not reported or not known = –; not applicable, NA.

As a result of the campaign, a significant uplift in website users (x10) and events (x5) was seen during the campaign vs. the 4 weeks prior (Table 1). A direct word search was the leading method used by the general population to get to the campaign website. The number of users and website events continued to grow even 1 month after the campaign had finished (users x13 and events x7 compared to website use 4 weeks prior to the campaign).

### Outcome evaluation (short term)

A total of 661 WA respondents took part in the campaign outcome evaluation. As shown in Table 2, respondents were representative of the WA population in terms of gender, age group, and location. Whether calculated as a proportion of total respondents or sub-set proportion of the cognitive hierarchy, one in four respondents (24%) were aware of the *Good Arts, Good Mental Health*^®^ message (Table 3). As shown in Table 2, total awareness (unprompted plus prompted awareness) did not differ by gender or age group; however, people in the metropolitan area were significantly more likely to be aware of the message than those in regional areas (*p* < 0.02). Of those aware of the message, most indicated that they saw/heard the message via social media. Whether calculated as a proportion of total respondents or as a proportion within the cognitive hierarchy (i.e., those who were aware), comprehension of the message was 49% (Table 3). As a proportion of total respondents, agreement with the message was 89%. However, in terms of the cognitive hierarchy, of those who were aware and comprehended the message, agreement was 97%. As a proportion of total respondents, intention to act on the message was 42%; however, in terms of the cognitive hierarchy, of those who were aware, comprehended, and agreed with the message, intention to act was 72%. Overall, agreement and intention to act on the message significantly increased with age, while males were significantly less likely to comprehend, agree, or to form an intention to act on the message (Table 2).

**Table 2:** Survey respondent demographic (*n* = 661^*^).

**Variable**	**Level**	***n***	**%**	**2021 Western Australian population% (77, 78)**	**Proportion of total respondents**			
					**Awareness**	**Comprehension**	**Agreement**	**Intention**
Gender	Female	371	56%	50%	20%	55%	94%	52%
	Male	283	43%	50%	28%	41%	83%	28%
	Non-binary	7	1%	Not reported	29%	71%	100%	57%
					*p* = NS	*p* < 0.001	*p* < 0.001	*p* < 0.001
Age Group^*^	18–29 years	154	23%	23%	29%	46%	84%	33%
	30–49 years	309	47%	45%	24%	49%	90%	42%
	50–65 years	198	30%	32%	20%	53%	92%	47%
					*p* = NS	*p* = NS	*p* = 0.04	*p* = 0.03
Location	Metropolitan	514	78%	79%	26%	48%	88%	41%
	Regional	147	22%	21%	16%	52%	93%	43%
					*p* = 0.02	*p* = NS	*p* = NS	*p* = NS

* NB 18–65 years only (17 years and under, 66 years, and over excluded as not part of the campaign target group).

**Table 3:** Cognitive impact of the GAGMH media campaign by (a) total respondents *n* = 661^*^ and (b) sub-set analysis of each preceding level in the cognitive impact hierarchy *n* = 157.

**a. Proportion of total respondents**				**b. Cognitive impact hierarchy sub-set** ***(proportion aware followed by subset analysis of each preceding level in the hierarchy)***		
**Variable**	**Level**	***n***	**%**	**Variable**	***n***	**%**
Awareness	No	504	76	Awareness *(of those surveyed)*	**157**	24
	Yes	**157**	24			
Comprehension	No	337	51	Comprehension *(of those aware)*	77/157	49
	Yes	324	49			
Agreement	No	72	11	Agreement *(of those aware and who comprehended)*	75/77	97
	Yes	589	89			
Intention	No	387	58	Intention (*of those aware, comprehended and who agreed)*	54/75	72
	Yes	274	42			

* NB 18–65 years only (17 years and under, 66 years and over excluded as not part of the campaign target group).

## Discussion

Since the 1980s, public health professionals in Australia have adopted many of the concepts and tools of commercial marketers including the use of large scale media campaigns to promote a health-related message (33). By documenting the *Good Arts, Good Mental Health*^®^ media campaign and evaluation, this paper contributes to the public health, mental health, and arts-health literature as it increases knowledge of and provides insight about *how to plan, implement, and evaluate* an arts-mental health promotion media campaign for the general population. Mass media campaigns that encourage members of the general population to “shift” their behavior by encouraging actions that enhance wellbeing is a common health promotion strategy as this approach, at a population level, can benefit more people (overall) than targeting only specific groups within the population (35).

### Process evaluation

Attempting to influence population-level thinking about arts engagement as a strategy for promoting and maintaining mental wellbeing requires interventions that can reach a large number of people at a relatively low cost (34). Overall, our campaign budget of AUD$198,965 was very small compared to other population-level health promotion campaigns (e.g., 2015 Make Healthy Normal AUD$3.5Million (6) and 2008–10 Find Thirty AUD$1.8Million). Despite our budget limitations, and lack of TV advertising, the process evaluation results suggest that the GAGMH campaign (Wave 1) was successfully implemented. As mentioned above, to achieve optimal population-level brand awareness, our “reach” goal was 36%−46% and our “frequency” goal was two to five exposures (68–72). Given this, our advertising reach can be rated as “good” given that we reached a minimum of 33% of the WA population, up to a maximum of 80% (if reach by channel/platform was taken as mutually exclusive). In comparison, campaign frequency could be considered as “very good” with an average of 7.3 across channels/platforms. If we err on the side of caution and assume our reach was 33%, to maximize reach in future waves of the campaign, it is suggested that a “frequency cap” at five exposures be implemented for Meta, Spotify, and radio. While not used in Wave 1 of the campaign, a frequency cap (79) would control how many times the target audience sees an arts-mental health advert during the campaign. With regard to effectiveness, a “good” cost-per-reach was suggested to be between $0.20 and $1.00 (73). In comparison, the GAGMH campaign cost-per-reach was found to be “very good” with an average cost-per-reach of $0.05, with the best cost-per-reach of $0.02 delivered by Meta. As social media is ingrained in our daily lives, with high usage and ease of engagement, it is an attractive tool for health behavior communication efforts and promoting behavior change (80). As our findings suggest social media was the most effective method for promoting mental wellbeing via the arts, priority will be given to social media campaign investment for future waves of the campaign.

As a result of the success of the GAGMH campaign implementation, a significant uplift in website users and events was observed. It is interesting to note that even though Meta and Spotify had clickable, direct links to the GAGMH website, a direct word search via a search engine was the leading method used by the general population to access the GAGMH website. The general population tends to search for health information through search engines due to its convenience, accessibility, and decentralized nature (81). In comparison, reasons for social media clicks include enjoyment, incentives, pleasing others, and passing time, while non-clicks resulted from factors including lack of motivation, lack of time, technical constraints, not wanting to “train the algorithm” to display similar content, and privacy concerns (82). In terms of website direct word searches, the results of our study should be seen positively as radio and social media adverts are often a conversation starter that spark more in-depth interactions (e.g., web searches) (82). When this occurs, this could be seen as a more authentic signal of relational and cognitive investment in a campaign than interaction with one-click social media links (82).

### Campaign outcomes

At a population level, public health media campaigns are an important strategy for informing large numbers of people about health research/evidence and health recommendations. Public health media campaigns are also essential for creating a strong social environment to encourage, enable, and reinforce a promoted message or to focus on the benefits (e.g., mental wellbeing) of community members adopting suggested health behaviors (i.e., arts engagement) (34, 83, 84). Our goal for the GAGMH campaign (Wave 1) was to achieve WA general population *awareness* between 4% and 30% (76). This was achieved. Whether calculated as a proportion of total respondents or sub-set within the cognitive hierarchy, one in four respondents (24%) were aware of the *Good Arts, Good Mental Health*^®^ tagline. It should be noted that most respondents indicated they saw/heard the message via social media—this result also fits with our process evaluation results which found that the highest reach (948,000 people) was delivered by Meta. However, as outlined above, it is interesting that website visits mostly occurred via a direct word search. While awareness did not differ by gender or age group, respondents in the metropolitan area were significantly more likely to be aware of the message than those in regional areas. Possible explanations as to why this occurred include (1) scheduling and (2) differences in the promotion of visual, audio, and static assets across channels (1). Overall campaign scheduling focused on high-rating morning, drive-home, and weekend radio segments and popular social media and Spotify user times. In the metropolitan area, there was high contract compliance and engagement with radio stations/talent, whereas in some regional areas, scheduled “live radio reads” did not occur and possibly impacted awareness. As our advertising was scheduled during “peak times” when the media environment is crowded with other advertising messages (1), this too may have impacted awareness overall and in regional areas. There is also the possibility that differences in the promotion of campaign assets across channels impacted awareness. This is likely due to a higher proportion (79%) of the advertising budget being spent on the metropolitan area as this is where most of the WA population resides. In addition, a distribution company was hired to put up signage in the metropolitan area; however, due to distance and associated costs, this was not possible for regional areas. Future GAGMH campaigns should make a concerted effort to increase the regional reach of the message by directing a higher proportion of the advertising budget to target regional areas (e.g., via social media, local radio, and signage).

Established WA health promotion campaigns (with TV advertising) have been found to achieve average *comprehension* levels of 43% of total respondents surveyed or 74% as a proportion of the cognitive hierarchy (i.e., the proportion of those who were aware and comprehended the message) (48). When compared to the literature average (43%) for total respondents, our campaign comprehension of 49% was higher than other WA health promotion campaigns. However, when compared to the literature average (74%) within the cognitive hierarchy, our message comprehension (49%) was found to be much lower. A possible explanation for comprehension of “other” WA health promotion messages being higher is due to years of prior exposure via media campaigns (that include TV advertising), event sponsorships, access to resources, learning, and engagement opportunities (48). In terms of the GAGMH campaign, this suggests that work still needs to be continued in terms of advertising, and at a ground level (sponsorships, resources, learning, and engagement opportunities) to increase both awareness and understanding of the GAGMH tagline and manifesto. It should be noted that when the open-ended comprehension responses were coded, of those respondents who were “aware but who did not comprehend the message,” this was because most responses related to art therapy/therapies instead of recreational arts (see above definition for why this is different) or focused on mental illness rather than promoting, maintaining, and/or improving mental health/mental wellbeing. Before future campaigns are run, this outcome should be discussed with project partners to co-design solutions such as the stronger promotion of recreational arts definitions, distinctions, and arts activity options and to increase understanding of the arts-mental wellbeing (vs. illness) relationship.

As outlined above, as this was the first time the GAGMH media campaign had been run, after the comprehension questions, all respondents were informed: “*Good Arts, Good Mental Health*^®^ *aims to encourage people to do arts activities that makes them feel good for better mental wellbeing. This includes reading books, listening to music, playing a musical instrument, singing, dancing, going to a concert, woodwork, craft, sewing, pottery, painting, creative writing, coloring, and so much more*.” Respondents were then asked if they agreed with the message and if they intended to do something about the message. Established WA health promotion campaigns (that include TV advertising) have been found to achieve average *agreement* levels of 40% of the total respondents surveyed or 92% as a proportion of the cognitive hierarchy (i.e., the proportion of those who were aware, comprehended, and agreed with the message) (48). In comparison, general population agreement with our campaign for total respondents was 89%, and within the cognitive hierarchy it was 97%. When compared to the literature average for total respondents, the general population agreement with our campaign more than doubled that achieved by other WA health promotion campaigns. In addition, general population agreement within the cognitive hierarchy was also higher than that achieved on average by other WA health promotion campaigns. This likely indicates that the WA population is supportive of the GAGMH campaign. Given the campaign was co-designed in a respectful manner with the WA community and other project partners, this finding is not surprising, as a tagline and manifesto that is positive, solution focused, and designed by community, is more likely to translate, resonate, and be accepted by community (51, 85).

*Intention* to act on the GAGMH message was 42% of total respondents and 72% within the cognitive hierarchy (i.e., proportion who were aware, comprehended, agreed, and intended to act on the message). General population intention to act resulting from the GAGMH campaign was double the average intention received by other WA health promotion campaigns reported in the literature (16% total respondents and 41% cognitive hierarchy) (48). A possible reason for this is that positively framed messages that provide reasons, ways, increase confidence, and encourages people to “*try”* have been found to be effective (39). If reported intentions translate into behavioral action whereby more WA community members are engaging in the arts for their mental wellbeing, this is a particularly positive outcome for individual and community mental health. In addition, this is also a positive outcome for WA artists and arts organization, in terms of the potential for increased attendance, income, perceived community value of the arts, and artist wellbeing. It should be noted that for this campaign, agreement and intention to act on the message significantly increased with age, which is consistent with the literature for WA health promotion messages (48). In addition, males were significantly less likely to comprehend, agree, or to form an intention to act on the GAGMH message. The literature also suggests that males are less likely than females to adopt health enhancing behaviors or to recognize and seek help for mental health issues (86–88). Given this, to increase GAGMH translation among males and younger people, qualitative and quantitative research should be conducted with these target audiences to increase campaign effectiveness and inclusivity. In particular, research should be conducted to better understand whether or not GAGMH campaign assets appealed to them, if they have any specific preferences regarding imagery, language (e.g., humor, positive/negative words, solution/problem focused, and authenticity of the communication), mode of delivery (e.g., social media, radio, signage), and communication intensity (e.g., 15 or 30 second ads/reels), and if there are gender or age-specific barriers to arts engagement that need to be addressed (e.g., stereotypes, accessibility, location, cost, attitudes, beliefs, knowledge/skills, time, etc).

### Good Arts, Good Mental Health (Wave1) was successful

**Figure 5:**
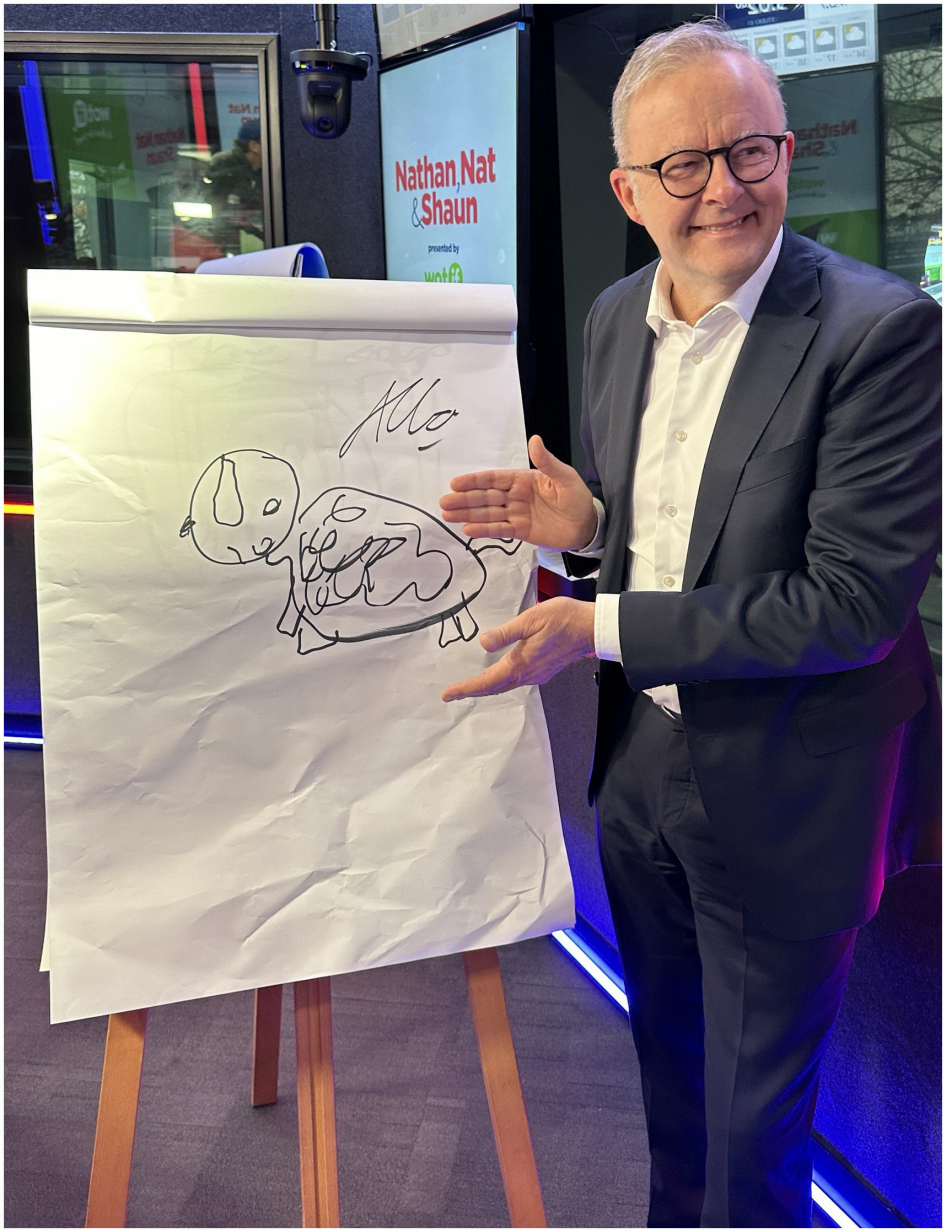
The Australian Prime Minister, the Hon. Anthony Albanese taking part in a GAGMH drawing challenge with Nathan, Nat and Shaun on Nova 93.7 radio (Note: the drawing is of the Prime Ministers dog ‘Toto'). Photo Credit and Permission: Nova 93.7.

Overall, GAGMH was successful as a population-level, arts-mental health promotion message, with process and outcome targets achieved. In fact, the implementation and outcome results achieved were far beyond the expectations of the GAGMH team given our small advertising budget and the (land) size of Western Australia. A number of factors may have contributed to the success of the campaign. First, the collaboration between the GAGMH team, our project partners, and service providers was mutually respectful, inclusive, openly adapted to feedback, and came from a place of shared values toward empowering the general population to improve their mental wellbeing via the arts (51). Our project partners willingly assisted in the translation of our organic media (e.g., via posts/reposts, inclusion in their newsletters, and assistance with signage distribution) and our paid media via their channels which resulted in reach beyond what the GAGMH team could have achieved ourselves. Our service providers also delivered bonus, pro-bono value of AUD$90,154 to the campaign via the provision of free radio advertising spots and social media posts. Show and talent integration by radio stations was also above expectations, for example, the breakfast team of one of the radio stations designed a fun and inclusive “GAGMH drawing challenge” that included the community, celebrities, sports stars, and in September 2024, the Australian Prime Minister, the Hon. Anthony Albanese MP (Figure 5).

Second, as suggested in the literature (46, 51, 89), formative research with partner organizations and the targeted audience was used to co-design the campaign development and was of particular benefit in guiding the selection of campaign assets, channels, imagery, language, and the distillation of research into a single clear message. We carefully defined the campaign target group and, when conducting our formative research, aimed to see “solutions” from the perspective of the target group, rather than from our role as arts-health research academics (51). To encourage behavior change, the creation of the GAGMH message and manifesto focused on target group lifestyles, budgets, attitudes, choice, mental wellbeing, helpful frames, and deeply held values (e.g., hedonism, stimulation, self-direction, and universalism) (51, 85). GAGMH's message and manifesto also aligned with a story structure that was positively framed and solution focused, rather than negative, fear, guilt, or problem focused (85). We presented a positive outcome (e.g., mental wellbeing, “*good mental health”*) and addressed the issue of people thinking they needed a high level of artistic skill, talent, ability, or expertise to take part—this was highlighted in our formative research as a major barrier that undermine engagement in the arts. We addressed this in the manifesto by stating “*you don't have to be good at art for the arts to be good for you.”* The GAGMH media campaign also empowered people to take action to achieve the promoted outcome (i.e., “*do the arts activity that makes you feel good, try for two hours per week”*). Importantly, the campaign acknowledged that every time a person is exposed to an issue related message, it is strengthened as a frame in their mind and therefore more likely to influence behavior by being activated the next time they think about that issue (85). Focusing therefore on negative myths or concepts (e.g., people thinking they are “bad” at art *and* people thinking they have to be an “expert” to do art) would have had the counterproductive effect of activating and strengthening the negative myths and concepts that GAGMH is trying to dispel. Third, the GAGMH campaign followed the “4 Ps” of marketing. That is, we made sure the right “Product” (i.e., the arts activity a person has a preference for and that makes them feel good) was available at the right “Price” (i.e., low cost, no cost activities promoted; arts dose/time suggested), in the right “Place” (i.e., community suggested channels, GAGMH website, and GAGMH social media), and was well “Promoted” (90). In addition, we also emphasized the mental wellbeing benefit (product benefit) of the arts rather than only promoting engagement in the arts (e.g., participation and attendance at events). For example, the social media tile for “reading” (see Figure 3) includes an image of someone reading, the word reading, but also highlights an evidence-based, mental wellbeing benefit of reading (i.e., “*reading makes me feel relaxed*”) (83).

### Strengths and limitations of this study

As Western Australia is representative of the broader Australian population (e.g., gender distribution, 5-year age groups, median age, population over 15 years, proportion born in Australia, household composition, average household size, employment, etc) (91), a strength of this study is that the findings are applicable and can be generalized to other Australian states/territories. In addition, the GAGMH campaign offers a promising model for population-level, mental health promotion in other countries, with similar health and socio-demographic profiles to Australia (e.g., New Zealand, Canada, United Kingdom, and various member states within the European Union) who are also facing similar mental health challenges in their general population. Other strengths of this study include the evaluation data being collected by an independent market research company, therefore reducing the possibility of researcher bias, and all open-ended responses being double-coded. Limitations of the evaluation reported in this study include the outcome data being self-reported and therefore subject to recall bias. Data on actual behavior change resulting from reported intentions were also not collected and therefore cannot be commented on but should be collected in future studies or waves of the campaign. In addition, our respondent comparisons only occurred in relation to gender, age group, and location. To better understand and increase campaign effectiveness and inclusivity, future studies or waves of the GAGMH campaign should consider extending the data collection and analysis to include other important demographic and effect modifying variables (e.g., education and income).

## Conclusion

Population-level health communication via media campaigns aim to transmit public health concepts to large numbers of people and if implemented correctly can result in meaningful changes in community awareness, attitudes, and health behaviors (32). As longer running and more intensive campaigns are likely to be more effective than a single, one-off campaign (40), it is recommended that funding be sought so that a second wave of the GAGMH campaign can be run within the next 12 months. Future waves of the GAGMH campaign should aim to reinforce campaign awareness and also increase understanding of GAGMH concepts, focus more on the “arts-mental health dose,” and extend the evaluation by measuring behavioral action. Future campaigns should promote activities (e.g., singing, reading, dancing), events (e.g., concerts, art exhibitions), and services (e.g., libraries, art classes, programs, workshops) and encourage arts-mental wellbeing policy and enviro-structural changes (e.g., allocated spaces and places for community arts to occur) (34).

Given that globally, mental health issues are increasing, the GAGMH campaign offers a promising model for population-level, arts-mental health promotion in Western Australia, other Australian states, and many other countries. The information contained in this study is useful to Public Health, Mental Health, and Arts-Health professionals in the planning, implementation, and evaluation of future population-level, arts-mental health promotion strategies, programs, and media campaigns. It is our view that there is substantial room for further arts-mental health research, evaluation, and translation to occur that explores and promotes the potential of the arts to positively impact community mental wellbeing.

## Data Availability

The datasets presented in this article are not readily available because secondary analysis of the data was not previously agreed to by respondents. Requests to access the datasets should be directed to A/Prof Christina Davies, christina.davies@uwa.edu.au.
